# Sex and *APOE* ɛ4 modify the effect of cardiovascular risk on tau in cognitively normal older adults

**DOI:** 10.1093/braincomms/fcac035

**Published:** 2022-02-18

**Authors:** Amaryllis A. Tsiknia, Emilie Reas, Katherine J. Bangen, Erin E. Sundermann, Linda McEvoy, James B. Brewer, Steven D. Edland, Sarah J. Banks, for the Alzheimer’s Disease Neuroimaging Initiative

**Affiliations:** 1 Department of Neurosciences, University of California, San Diego, La Jolla, CA, USA; 2 Research Service, VA San Diego Healthcare System, San Diego, La Jolla, CA, USA; 3 Department of Psychiatry, University of California, San Diego, La Jolla, CA, USA; 4 Department of Radiology, University of California, San Diego, La Jolla, CA, USA; 5 Department of Family Medicine and Public Health, University of California, San Diego, La Jolla, CA, USA

**Keywords:** Alzheimer’s disease, apolipoprotein E, cardiovascular disease risk, sex differences tau pathology

## Abstract

The interaction between *APOE* ɛ4 and vascular risk factors on cognitive function is stronger in women than in men. These effects may be mediated by the amount of tau pathology in the brain. Therefore, we examined whether *APOE* ɛ4 and sex modify cross-sectional associations between cardiovascular risk and tau deposition in cognitively normal older adults from the Alzheimer’s Disease Neuroimaging Initiative. We calculated the Framingham Heart Study cardiovascular disease risk score for 141 participants (74 women, 47 *APOE* ɛ4 carriers) with complete medical history data, processed tau-PET data and a Clinical Dementia Rating global score of 0.0 at the time of the tau-PET scan, implying no significant cognitive or functional impairment. We used linear regression models to examine the effects of sex, *APOE* ɛ4, cardiovascular risk and their interactions on tau deposition in the entorhinal cortex, inferior temporal cortex and a composite meta-region of interest of temporal lobe areas. We found a significant three-way interaction among sex, *APOE* ɛ4 status and cardiovascular disease risk on tau deposition in the entorhinal cortex (*β* = 0.04; 95% CI, 0.01–0.07; *P* = 0.008), inferior temporal cortex (*β* = 0.02; 95% CI, 0.0–0.05; *P* = 0.029) and meta-region (*β* = 0.02; 95% CI, 0.0–0.04; *P* = 0.042). After stratifying by *APOE* ɛ4 status to examine interactions between sex and cardiovascular disease risk on tau in *APOE* ɛ4 carriers and non-carriers, we found a significant two-way interaction between sex and cardiovascular disease risk on tau in the entorhinal cortex (*β* = 0.05; 95% CI, 0.02–0.08; *P* = 0.001), inferior temporal cortex (*β* = 0.03; 95% CI, 0.01–0.05; *P* =0.009) and meta-region (*β* = 0.02; 95% CI, 0.01–0.04; *P* = 0.008) only among *APOE* ɛ4 carriers. In analyses stratified by sex, higher cardiovascular risk scores were associated with higher levels of tau in the entorhinal cortex (*β* = 0.05; 95% CI, 0.02–0.08; *P* = 0.002), inferior temporal cortex (*β* = 0.02; 95% CI, 0.0–0.05; *P* = 0.023) and meta-region (*β* = 0.02; 95% CI, 0.01–0.04; *P* = 0.013) in female *APOE* ɛ4 carriers but not in male carriers. Our findings suggest that cognitively normal older women carrying at least one *APOE* ɛ4 allele, may be particularly vulnerable to the effects of cardiovascular disease risk on early tau deposition.

## Teaser

Tsiknia *et al*. report that among cognitively normal older adults, sex and *APOE* ɛ4 modify associations between cardiovascular disease risk and cortical tau deposition. Cognitively normal older women carrying at least one *APOE* ɛ4 allele, may be particularly vulnerable to the effects of cardiovascular disease risk on cortical tau deposition.

## Graphical Abstract

**Graphical Abstract:**
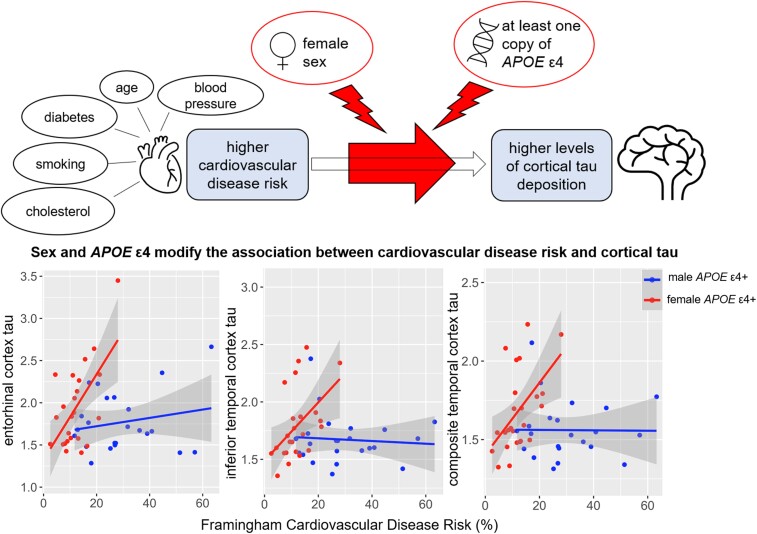

## Introduction

While it is clear that vascular risk factors^1,2^ and vascular neuropathology^2^ are associated with the clinical diagnosis of Alzheimer’s disease, some investigators have also proposed that vascular risk factors may be aetiologically relevant to Alzheimer’s disease neuropathology.^3^ Risk factors such as hypertension can contribute to brain hypoperfusion and blood–brain barrier damage, potentially facilitating amyloid and tau accumulation in the brain.^4^ Growing evidence from animal^5^ and human^6–9^ studies indicates that vascular risk burden is associated with increases in tau pathology, while a few studies report associations between vascular risk and amyloid accumulation only,^10^ or both amyloid and tau pathology.^11^ One group found that tau deposition mediated associations between cerebrovascular disease and cognitive impairment,^12^ and another study showed that tau burden mediated associations of cerebral blood flow and a marker of pericyte injury with cognitive scores.^13^

Studies have also shown that the impact of cardiovascular risk burden on cognitive decline and Alzheimer’s disease progression is stronger among carriers of the apolipoprotein ɛ4 (*APOE* ɛ4) allele.^14–16^*APOE* ɛ4, the major genetic risk factor for Alzheimer’s disease, cannot only promote Alzheimer’s disease pathogenesis directly by reducing amyloid clearance and increasing tau phosphorylation,^17–20^ but can also indirectly impact Alzheimer’s disease pathogenesis by increasing cardiovascular risk factors such as elevated plasma low-density lipoproteins, which are also risk factors for Alzheimer’s disease.^21^ A recent study of dementia free older adults demonstrated that higher vascular risk was associated with a stronger effect of *APOE* ɛ4 on memory decline in women but not men.^22^ Although these findings are consistent with evidence of a more robust *APOE* ɛ4 effect on clinical outcomes^23^ and tau pathology^24–27^ in women, the mechanism for a stronger *APOE* ɛ4 effect in women remains unknown.

Sex differences in the effect of cardiovascular burden on brain health could constitute a strong candidate mechanism. Our group previously found that among cognitively normal (CN) older adults aged between 56 and 75 years, women displayed stronger associations between higher pulse pressure, a surrogate marker of arterial stiffness, and white matter microstructural abnormalities, compared with men in the same age group.^28^ We also previously demonstrated that among a cohort of community-dwelling older adults with a mean age of 67 years, higher cardiovascular risk was associated with greater executive function and memory decline among women but not men, despite higher risk scores among men.^29^ Similarly, another group found that among older adults between 60 and 100 years of age, higher systolic blood pressure was correlated with worse cognitive performance among hypertensive women but not men.^30^ Given the strong link between tau pathology and clinical presentation of Alzheimer’s disease,^31,32^ the exacerbated effects of cardiovascular risk on cognition in women could be related to tau pathology. Therefore, we examined the role of sex and *APOE* ɛ4 status in modifying the effect of cardiovascular disease risk on cortical tau in CN older adults.

## Materials and methods

### Participants

All data used in preparation of this manuscript were obtained from the Alzheimer’s Disease Neuroimaging Initiative (ADNI) database (accessed from ida.loni.usc.edu in April 2021). ADNI is a study which aims to measure the progression of MCI and early Alzheimer’s disease using multi-modal imaging methods. ADNI inclusion criteria have been described previously (refer to http://adni.loni.usc.edu/). Notably, individuals with Hachinski ischaemic scores >4 at screening are excluded from ADNI. The Hachinski Ischaemic score is a widely used questionnaire designed to distinguish individuals with vascular dementia from individuals with pure Alzheimer’s disease and mixed dementia (i.e. dementia caused by both Alzheimer’s disease and vascular pathology).^33^ Additional inclusion criteria for our study included fully processed regional Flortaucipir PET (FTP-PET) for measurement of tau deposition, clinical and medical history data required for cardiovascular disease risk calculations, as well as a Clinical Dementia Rating global score (CDR-GS) equal to 0.0 at the time of FTP-PET scan.

### Clinical assessment

We used the participants’ Clinical Dementia Rating scale (CDR) score to determine clinical status at the time of FTP-PET scan.^34^ This was done to avoid potential sex-related bias from using ADNI-determined diagnoses, which rely on verbal memory tests in which women frequently outperform men despite similar levels of Alzheimer’s disease pathology.^35–38^ We obtained CDR-GS measured either at the same study visit as the FTP-PET scan or at the study visit closest to the FTP-PET scan (average time difference between CDR and FTP-PET measurement was 52 days). Participants with a CDR-GS equal to 0.0 were included.

### Cardiovascular disease risk assessment

We quantified cardiovascular disease risk using the Framingham Heart Study cardiovascular disease (FHS-CVD) risk algorithm—a well-validated tool that has been used in both research and primary care.^39,40^ The FHS-CVD risk score is a sex-specific measure of cardiovascular disease risk accounting for age, systolic blood pressure, anti-hypertensive treatment, total cholesterol, high density lipoprotein cholesterol, self-reported history of diabetes and current smoking status.^39^ The risk score represents the probability of cardiovascular events (such as coronary death, myocardial infarction, etc.) occurring within 10 years of risk assessment. We chose the FHS-CVD risk measure obtained closest to the FTP-PET scan with the restriction that the period of time between FHS-CVD assessment and FTP-PET scan could not be more than 10-years. This criterion was applied to ensure this time lag did not exceed the 10-year predictive window of the cardiovascular risk assessment.

### *APOE* genotyping

*APOE* genotype data were available for all participants and was obtained from the LONI database. Details regarding blood sample collection and genotyping procedures for ADNI can be found in adni.loni.usc.edu. Homozygotes and heterozygotes for the ɛ4 allele (including ɛ2/ɛ4, ɛ3/ɛ4 and ɛ4/ɛ4 genotypes) were combined into a single group and categorized as *APOE* ɛ4 carriers, while participants with ɛ2/ɛ3 or ɛ3/ɛ3 genotypes were categorized as *APOE* ɛ4 non-carriers. There were no ɛ2 homozygotes in our study sample.

### Brain imaging acquisition and processing

We downloaded Aβ and tau-PET data (from ida.loni.usc.edu, accessed in April 2021) which Landau and colleagues had previously processed at the University of California, Berkeley. ADNI brain imaging acquisition and processing procedures have been described in detail elsewhere http://adni.loni.usc.edu/methods/documents. Briefly, structural MRI scans are performed on 3T scanners using either a 3D MPRAGE or IR-SPGR T_1_-weighted sequence with sagittal slices and spatial resolution of 1.1 × 1.1 × 1.2 mm^3^. Structural MRI scans are then skull-stripped, segmented and parcellated using the FreeSurfer (version 5.3.0; http://surfer.nmr.mgh.harvard.edu). Aβ PET images are acquired 50–70 min post Florbetapir injection in a series of four 5-min frames and tau-PET images are acquired 75–105 min after FTP injection in a series of six 5-min frames. After the raw PET data are assessed for quality, each of the acquired frames is extracted and co-registered to the first frame to account for subject motion. The motion-corrected dynamic image set is then averaged and smoothed to a uniform isotropic resolution of 8 mm full width at half maximum and then co-registered with the subject’s processed structural MRI. Standardized uptake value ratios (SUVRs) of FTP uptake are computed for each FreeSurfer-derived region by referencing to the mean cerebellar grey matter deposition.^41^ To determine Aβ positivity we used the SUVR of a cortical summary region, which is intensity normalized by a whole-cerebellum FreeSurfer region. An SUVR cut-off of 1.11 was used to determine Aβ positivity.^42,43^ Lastly, regional FTP data are corrected for partial-volume effects using the Geometric Transfer Matrix approach.^44,45^ To examine regional tau burden, we used a bilateral volume-weighted composite of the entorhinal cortex (EC) and the inferior temporal cortex (ITC), given that these regions are cortical sites of early Alzheimer’s disease-related tau deposition.^46–48^ Additionally, we examined tau deposition in a larger and likely more stable composite region of interest (meta-ROI) of tau deposition, consisting of a volume-weighted average of the bilateral amygdala, fusiform gyrus, middle temporal cortex, EC and ITC.

### Statistical analyses

All statistical analyses were computed with the R (version 4.0.3). Differences in demographic and clinical variables by sex and *APOE* ɛ4 status were assessed using Welch’s independent *t*-tests for continuous variables and Fisher’s exact tests for categorical variables. Next, we applied a series of linear regression models to examine the modifying role of sex and *APOE* ɛ4 on the association between FHS-CVD risk score and tau deposition covarying for the time lag between FHS-CVD risk measurement and FTP-PET scan. First, we used a linear regression model testing for a three-way interaction between sex, *APOE* ɛ4 status and FHS-CVD risk on tau. In the case of a significant three-way interaction, we stratified our sample by *APOE* ɛ4 status, and used a linear regression model testing for a two-way interaction between sex and FHS-CVD risk on tau in *APOE* ɛ4 carriers and *APOE* ɛ4 non-carriers separately. Finally, in the case of a significant two-way interaction between sex and FHS-CVD risk on tau, we further stratified by sex to examine the main effect of cardiovascular risk in men and women separately. Analyses were repeated with models additionally adjusted for age at FTP-PET scan, Aβ status and years of education to assess potential confounding effects.

### Data availability

Data used in preparation of this manuscript were obtained from the LONI database in April 2021 (ida.loni.usc.edu).

## Results

### Sex differences in demographic characteristics

**Figure 1:**
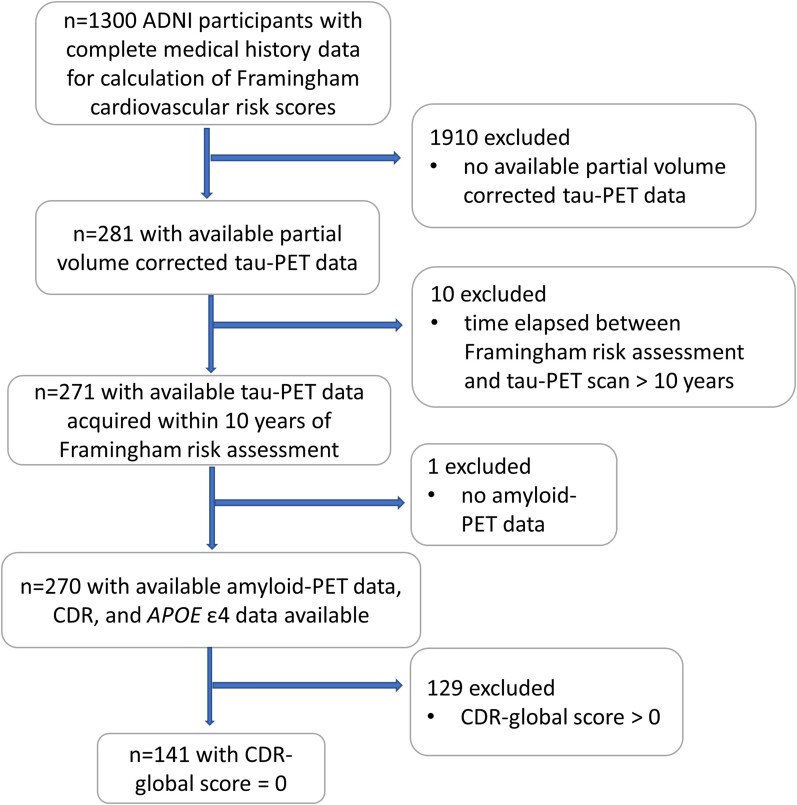
**Subject exclusion based on eligibility criteria and arrival at the final study sample**. ADNI = Alzheimer’s Disease Neuroimaging Initiative; CDR = Clinical Dementia Rating.

Of the sample of 141 participants who met our inclusion criteria (Fig. 1), 52% were women and 33% carried at least one *APOE* ɛ4 allele. Participants’ age at the time of cardiovascular risk assessment ranged between 55 and 90 years [mean (SD) = 72.1 (6.45)] and time lag between cardiovascular risk assessment and FTP-PET scan ranged between 2 and 10 years [mean (SD) = 4.94 (1.55)]. Women in our sample had fewer years of education (*P* < 0.05), lower FHS-CVD risk scores (*P* < 0.0001) and higher total and high density lipoprotein (HDL) cholesterol (*P* < 0.001 and *P* < 0.0001, respectively) than men. Among *APOE* ɛ4 carriers, women had lower FHS-CVD risk scores (*P* < 0.0001), were younger at the time of FHS-CVD risk assessment (*P* < 0.05) and had higher total and HDL cholesterol (*P* < 0.05 and *P* < 0.01, respectively) than men. Among *APOE* ɛ4 non-carriers, women had fewer years of education (*P* < 0.01), higher HDL cholesterol (*P* < 0.001) and lower FHS-CVD risk scores (*P* < 0.0001) than men. Details regarding demographic and clinical variables of our sample can be found under Table 1.

**Table 1:** Demographic and clinical sample characteristics

Variables	Total cohort (*n* = 141)		*APOE* ɛ4 carriers (*n* = 47)		*APOE* ɛ4 non-carriers (*n* = 94)	
	Women (*n* = 74)	Men (*n* = 67)	Women (*n* = 24)	Men (*n* = 23)	Women (*n* = 50)	Men (*n* = 44)
Aβ positive, *n* (%)	28 (37.8)	23 (34.3)	14 (58.3)	12 (52.2)	14 (28)	11 (25)
Education, years, mean (SD)	16.3 (2.44)	17.3 (2.47)	16.5 (2.38)	16.7 (2.85)	16.1 (2.48)	17.6 (2.21)
White, *n* (%)	70 (94.6)	63 (94)	23 (95.8)	22 (95.7)	47 (94)	41 (93.2)
African American or Black, *n* (%)	3 (4.1)	2 (3)	1 (4.2)	1 (4.3)	2 (4)	1 (2.3)
Asian, *n* (%)	0	2 (3)	0	0	0	2 (4.5)
More than one race, *n* (%)	1 (1.4)	0	0	0	1 (2)	0
FHS-CVD risk score, %, mean (SD)	12.1 (5.76)	26.7 (11.3)	12.1 (5.89)	29.6 (14.3)	12.1 (5.76)	25.1 (6.25)
Age, years, mean (SD)	71.7 (6.08)	72.6 (0.86)	69.0 (6.04)	73.4 (7.96)	73.0 (5.7)	72.1 (6.25)
Systolic blood pressure, mm Hg, mean (SD)	134 (14.1)	136 (14.8)	136 (14.9)	136 (13.9)	134 (13.8)	136 (15.4)
Total cholesterol, mg/dL,	197 (38.6)	181 (29.6)	207 (38.6)	181 (35.5)	192 (38)	182 (26.5)
mean (SD)						
HDL cholesterol, mg/dL,	64.6 (15.5)	53.5 (11.0)	67.1 (15.4)	53.3 (12.0)	63.4 (15.6)	53.6 (10.6)
mean (SD)						
Taking anti-hypertensive	15 (20.3)	11 (16.4)	6 (25)	7 (30.4)	9 (18)	4 (9.1)
medication, *n* (%)						
Diabetic, *n* (%)	6 (8.1)	8 (11.9)	2 (8.3)	4 (17.4)	4 (8)	4 (9.1)
Smokers, *n* (%)	1 (1.4)	3 (4.5)	0	1 (4.3)	1 (2)	2 (4.5)
Entorhinal cortex tau-PET SUVR, mean (SD)	1.82 (0.398)	1.65 (0.303)	1.94 (0.492)	1.77 (0.351)	1.76 (0.334)	1.58 (0.255)
Inferior temporal cortex tau-PET SUVR, mean (SD)	1.73 (0.282)	1.60 (0.172)	1.8 (0.309)	1.67 (0.212)	1.70 (0.265)	1.56 (0.134)
Meta-ROI temporal lobe tau-PET SUVR, mean (SD)	1.62 (0.229)	1.50 (0.150)	1.68 (0.257)	1.56 (0.185)	1.59 (0.210)	1.47 (0.119)
Time between FHS-CVD Assessment and tau-PET scan, years, mean (SD)	4.77 (1.55)	5.13 (1.55)	4.79 (1.82)	5.00 (1.21)	4.76 (1.42)	5.2 (1.71)

Mean and SD are provided for continuous variables and *n* and % are provided for categorical variables. Aβ = amyloid-β; FHS-CVD = Framingham Heart Study cardiovascular disease; HDL = high density lipoprotein; SUVR = standardized uptake value ratio

### Interaction effect of sex, *APOE* ɛ4 and FHS-CVD risk on tau

**Figure 2:**
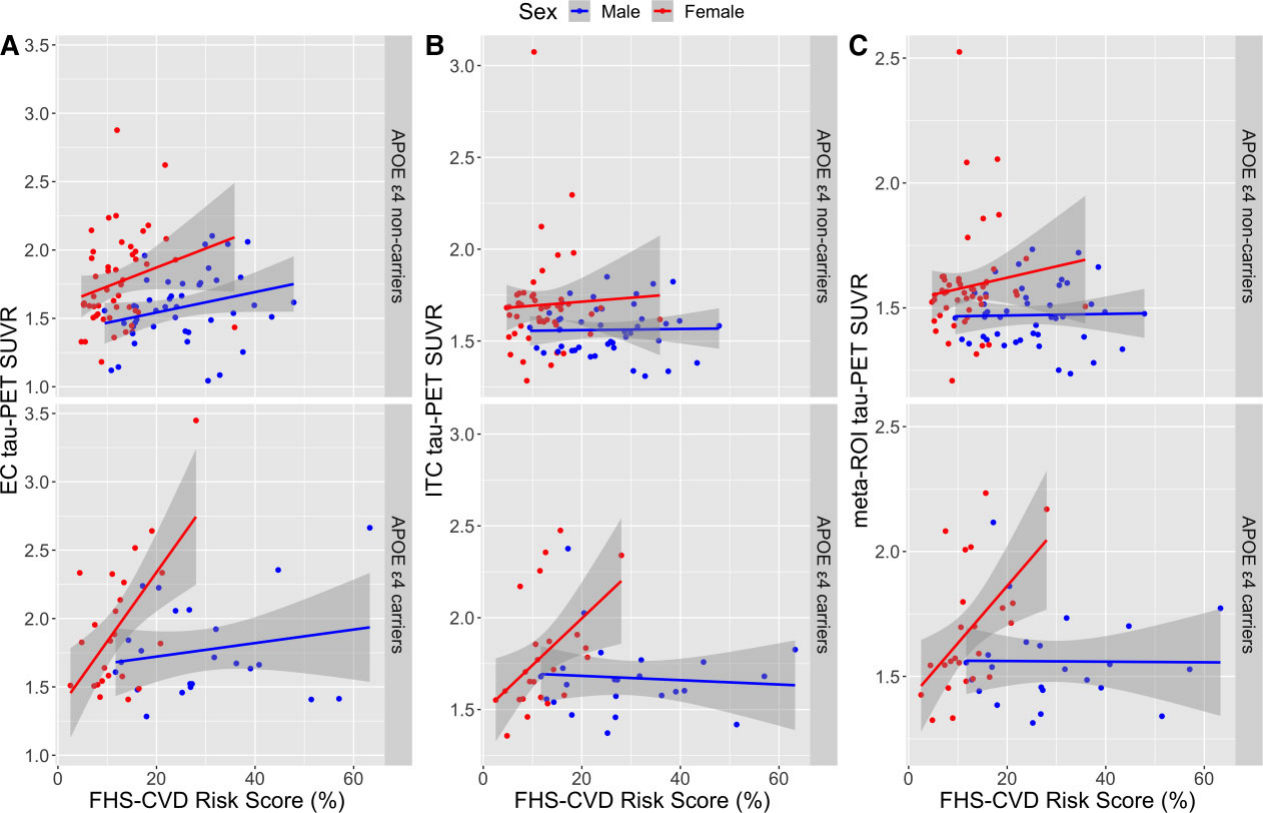
**Sex and *APOE* ɛ4 modify associations between cardiovascular disease risk and tau deposition**. Scatter plots depicting sex differences in associations between FHS-CVD risk score and tau deposition in the (**A**) entorhinal cortex, (**B**) inferior temporal cortex and (**C**) a composite temporal lobe meta-ROI, among *APOE* ɛ4 non-carriers (*top*) and *APOE* ɛ4 carriers (*bottom*). Shaded regions represent 95% confidence intervals. We found significant interactions between sex and FHS-CVD risk on tau deposition in the EC (*β* = 0.05; 95% CI, 0.02–0.08; *P* = 0.001), ITC (*β* = 0.03; 95% CI, 0.01–0.05; *P* = 0.009) and meta-ROI (*β* = 0.02; 95% CI, 0.01–0.04; *P* = 0.008) among *APOE* ɛ4 carriers (*bottom*) but not among non-carriers (*top*).

There was a significant three-way interaction among sex, *APOE* ɛ4 status and FHS-CVD risk on tau deposition in the EC (*β* = 0.04; 95% CI, 0.01–0.07; *P* = 0.008), ITC (*β* = 0.02; 95% CI, 0.0–0.05; *P* = 0.029) and meta-ROI (*β* = 0.02; 95% CI, 0.0–0.04; *P* = 0.042) (Table 2). To aid our interpretation of the three-way interaction between sex, *APOE* ɛ4 and FHS-CVD risk on tau deposition, we stratified by *APOE* ɛ4 status to examine two-way interactions between sex and FHS-CVD risk on tau in *APOE* ɛ4 carriers and non-carriers separately. We found a significant two-way interaction between sex and FHS-CVD risk on tau in the EC (*β* = 0.05; 95% CI, 0.02–0.08; *P* = 0.001) (Fig. 2A), ITC (*β* = 0.03; 95% CI, 0.01–0.05; *P* = 0.009) (Fig. 2B) and meta-ROI (*β* = 0.02; 95% CI, 0.01–0.04; *P* = 0.008) (Fig. 2C) only among *APOE* ɛ4 carriers (Table 3). Since two-way interactions between sex and FHS-CVD risk were significant only among *APOE* ɛ4 carriers, we further stratified by sex to examine main effects of FHS-CVD risk on tau in male and female *APOE* ɛ4 carriers separately. We found that higher FHS-CVD risk was associated with higher levels of tau in the EC (*β* = 0.05; 95% CI, 0.02–0.08; *P* = 0.002), ITC (*β* = 0.02; 95% CI, 0.0–0.05; *P* = 0.023) and meta-ROI (*β* = 0.02; 95% CI, 0.01–0.04; *P* = 0.013) in female *APOE* ɛ4 carriers but not male carriers (Table 4).

**Table 2:** Three-way interaction effect of *APOE* ɛ4, sex and cardiovascular risk on tau deposition

Predictor variables	EC tau-PET SUVR			ITC tau-PET SUVR			Meta-ROI tau-PET SUVR		
	Estimate	CI	*P*-value	Estimate	CI	*P*-value	Estimate	CI	*P*-value
FHS-CVD risk	0.01	−0.0 to 0.02	0.126	0.0	−0.01 to 0.01	0.939	0.0	−0.01 to 0.01	0.927
*APOE* (ɛ4+)	0.28	−0.14 to 0.70	0.187	0.15	−0.15 to 0.45	0.317	0.10	−0.15 to 0.34	0.425
Sex (female)	0.23	−0.12 to 0.58	0.195	0.12	−0.13 to 0.37	0.358	0.07	−0.14 to 0.27	0.525
Time lag	0.03	−0.0 to 0.07	0.059	−0.0	−0.03 to 0.02	0.913	−0.00	−0.02 to 0.02	0.920
*APOE* (ɛ4+) × FHS-CVD risk	−0.00	−0.02 to 0.01	0.563	−0.0	−0.01 to 0.01	0.781	−0.00	−0.01 to 0.01	0.927
Sex (female) × FHS-CVD risk	0.01	−0.01 to 0.02	0.539	0.00	−0.01 to 0.02	0.783	0.00	−0.01 to 0.02	0.477
*APOE* (ɛ4+) × sex (female)	−0.57	−1.12to−0.01	**0**.048*	−0.33	−0.73 to 0.06	0.099	−0.23	−0.55 to 0.10	0.175
*APOE* (ɛ4+) × sex (female) × FHS-CVD risk	0.04	0.01–0.07	**0**.008**	0.02	0.00–0.05	**0**.029*	0.02	0.00–0.04	**0**.042*

Results from linear regression analysis revealed a significant three-way interaction effect of *APOE* ɛ4, sex and FHS-CVD risk on tau deposition in the EC, ITC and meta-ROI after adjusting for the time between FHS-CVD risk assessment and tau-PET scan. Significant *P*-values are presented in bold, where *P <0.05, **P < 0.01, ***P < 0.005. CI = confidence interval; EC = entorhinal cortex; FHS-CVD risk = Framingham Heart Study cardiovascular disease risk; ITC = inferior temporal cortex; SUVR = standardized uptake value ratio.

**Table 3:** Two-way interaction effect of sex and cardiovascular disease risk on cortical tau deposition among *APOE* ɛ4 carriers but not non-carriers

Predictor variables	EC tau-PET SUVR			ITC tau-PET SUVR			Meta-ROI tau-PET SUVR		
	Estimate	CI	*P-*value	Estimate	CI	*P*-value	Estimate	CI	*P*-value
*APOE* ɛ4 carriers									
FHS-CVD risk	0.00	−0.01 to 0.01	0.553	−0.00	−0.01 to 0.01	0.753	−0.00	−0.01 to 0.01	0.968
Sex (female)	−0.36	−0.88 to 0.16	0.169	−0.22	−0.57 to 0.13	0.21	−0.16	−0.45 to 0.13	0.275
Time lag	0.06	−0.02 to 0.13	0.132	0.00	−0.05 to 0.05	0.965	0.00	−0.04 to 0.04	0.995
Sex (female) × FHS-CVD risk	0.05	0.02–0.08	**0**.001***	0.03	0.01–0.05	**0**.009**	0.02	0.01–0.04	**0**.008**
*APOE* ɛ4 non-carriers									
FHS-CVD risk	0.01	−0.00 to 0.02	0.105	0.00	−0.01 to 0.01	0.942	0.00	−0.01 to 0.01	0.925
Sex (female)	0.22	−0.10 to 0.54	0.176	0.12	−0.12 to 0.36	0.341	0.07	−0.13 to 0.26	0.503
Time lag	0.02	−0.02 to 0.06	0.236	−0.00	−0.03 to 0.03	0.863	−0.00	−0.02 to 0.02	0.894
Sex (female) × FHS-CVD risk	0.01	−0.01 to 0.02	0.490	0.00	−0.01 to 0.01	0.771	0.00	−0.01 to 0.01	0.418

Results from linear regression analysis on *APOE* ɛ4 stratified groups revealed a significant interaction effect of sex and FHS-CVD risk on tau deposition in the EC, ITC and meta-ROI only among *APOE* ɛ4 carriers, after adjusting for the time between FHS-CVD risk assessment and tau-PET scan. Significant *P-*values are presented in bold, where *P < 0.05, **P < 0.01, ***P < 0.005. CI = confidence interval; EC = entorhinal cortex; FHS-CVD risk = Framingham Heart Study cardiovascular disease risk; ITC = inferior temporal cortex; SUVR = standardized uptake value ratio.

**Table 4:** Main effect of cardiovascular disease risk on cortical tau deposition in female *APOE* ɛ4 carriers but not male carriers

Predictor variables	EC tau-PET SUVR			ITC tau-PET SUVR			Meta-ROI tau-PET SUVR		
	Estimate	CI	*P-*value	Estimate	CI	*P-*value	Estimate	CI	*P*-value
Female *APOE* ɛ4 carriers									
FHS-CVD risk	0.05	0.02–0.08	0.002***	0.02	0.00–0.05	**0**.023*	0.02	0.01–0.04	**0**.013*
Time lag	0.03	−0.07 to 0.13	0.511	−0.02	−0.09 to 0.04	0.475	−0.02	−0.08 to 0.03	0.349
Male *APOE* ɛ4 carriers									
FHS-CVD risk	0.00	−0.01 to 0.01	0.768	−0.00	−0.01 to 0.00	0.371	−0.00	−0.01 to 0.00	0.494
Time lag	0.12	−0.01 to 0.25	0.064	0.07	−0.02 to 0.15	0.107	0.07	−0.00 to 0.13	0.062

Results from linear regression analysis on sex-stratified groups of *APOE* ɛ4 carriers revealed a significant main effect of FHS-CVD risk on tau deposition in the EC, ITC and meta-ROI only among female *APOE* ɛ4 carriers, after adjusting for the time between FHS-CVD risk assessment and tau-PET scan. Significant *P-*values are presented in bold, where *P < 0.05, **P < 0.01, ***P < 0.005. CI = confidence interval; EC = entorhinal cortex; FHS-CVD risk = Framingham Heart Study cardiovascular disease risk; ITC = inferior temporal cortex; SUVR = standardized uptake value ratio.

The three-way interaction between *APOE* ɛ4, sex and FHS-CVD risk on EC tau deposition (*β* = 0.04; 95% CI, 0.01–0.07; *P* = 0.012) remained significant after additionally adjusting for age, Aβ status and education, though this effect was attenuated in the ITC (*β* = 0.02; 95% CI, −0.01–0.04; *P* = 0.058) and meta-ROI (*β* = 0.01; 95% CI, −0.01–0.02; *P* = 0.093) (Table 5). Notably, Aβ positive participants had higher levels of tau across all three regions, though the main effect of Aβ status on tau was more robust in ITC (*β* = 0.18; 95% CI, 0.10–0.26; *P* < 0.001) and meta-ROI (*β* = 0.15; 95% CI, 0.09–0.22; *P* < 0.001) compared with the EC (*β* = 0.13; 95% CI, 0.01–0.26; *P* = 0.030). *APOE* ɛ4 stratified analyses revealed a significant two-way interaction effect of sex and FHS-CVD risk on EC (*β* = 0.05; 95% CI, 0.02–0.08; *P* = 0.004), ITC (*β* = 0.02; 95% CI, 0.00–0.04; *P* = 0.049) and meta-ROI (*β* = 0.02; 95% CI, 0.00–0.03; *P* = 0.045) tau deposition among *APOE* ɛ4 carriers but not non-carriers, after additionally adjusting for age, Aβ status and education (Table 6). Aβ status had a consistent main effect on tau deposition in the ITC and meta-ROI among both *APOE* ɛ4 carriers and non-carriers. Finally, in sex-stratified analyses of *APOE* ɛ4 carriers we found that higher FHS-CVD risk scores were associated with higher levels of tau in the EC (*β* = 0.06; 95% CI, 0.03–0.09; *P* = 0.002) and meta-ROI (*β* = 0.02; 95% CI, 0.00–0.04; *P* = 0.028) among female but not male carriers (Table 7). The effect of FHS-CVD risk on ITC tau in female carriers was no longer significant (*β* = 0.02; 95% CI, −0.00 to 0.04; *P* = 0.052) after adjusting for age, education and Aβ status. Lastly, we observed no effect of age and education on tau deposition in any of the models.

**Table 5:** Three-way interaction effect of *APOE* ɛ4, sex and cardiovascular risk on tau deposition, after additionally adjusting for age, Aβ status and education

Predictor variables	EC tau-PET SUVR			ITC tau-PET SUVR			Meta-ROI tau-PET SUVR		
	Estimate	CI	*P*-value	Estimate	CI	*P*-value	Estimate	CI	*P*-value
FHS-CVD risk	0.01	−0.01 to 0.02	0.239	0.00	−0.01 to 0.01	0.793	0.00	−0.00 to 0.01	0.652
*APOE* (ɛ4+)	0.19	−0.24 to 0.61	0.390	0.01	−0.28 to 0.30	0.945	−0.03	−0.27 to 0.20	0.773
Sex (female)	0.25	−0.11 to 0.61	0.166	0.11	−0.14 to 0.35	0.392	0.05	−0.15 to 0.24	0.641
Age	0.00	−0.01 to 0.01	0.532	0.00	−0.01 to 0.01	0.643	0.00	−0.00 to 0.01	0.210
Time lag	0.02	−0.01 to 0.06	0.224	−0.01	−0.04 to 0.01	0.389	−0.01	−0.03 to 0.01	0.223
Aβ status (Aβ+)	0.13	0.01–0.26	**0**.030*	0.18	0.10–0.26	**<0**.001****	0.15	0.09–0.22	**<0**.001****
Education	0.01	−0.01 to 0.04	0.226	0.00	−0.01 to 0.02	0.600	0.00	−0.01 to 0.02	0.588
*APOE* (ɛ4+) × FHS-CVD risk	−0.00	−0.02 to 0.01	0.809	0.00	−0.01 to 0.02	0.687	0.00	−0.00 to 0.01	0.455
Sex (female) × FHS-CVD risk	0.00	−0.01 to 0.02	0.681	0.00	−0.01 to 0.01	0.911	0.00	−0.01 to 0.01	0.554
*APOE* (ɛ4+) × sex (female)	−0.50	−1.06 to 0.07	0.084	−0.23	−0.61 to 0.16	0.242	−0.11	−0.42 to 0.20	0.475
*APOE* (ɛ4+) × sex (female) × FHS-CVD risk	0.04	0.01–0.07	**0**.012*	0.02	−0.00 to 0.04	0.058	0.01	−0.00 to 0.03	0.093

Results from linear regression analysis revealed a significant three-way interaction effect of *APOE* ɛ4, sex and FHS-CVD risk on tau deposition in the EC, but not the ITC or meta-ROI after adjusting the time between FHS-CVD risk assessment and tau-PET scan, age, Aβ status and education. Significant *P-*values are presented in bold, where *P < 0.05, **P < 0.01, ***P < 0.005, ****P < 0.001. Aβ = amyloid-β; CI = confidence interval; EC = entorhinal cortex; FHS-CVD risk = Framingham Heart Study cardiovascular disease risk; ITC = inferior temporal cortex; SUVR = standardized uptake value ratio.

**Table 6:** Two-way interaction effect of sex and cardiovascular disease risk on cortical tau deposition among *APOE* ɛ4 carriers but not non-carriers, after additionally adjusting for age, Aβ status and education

Predictor variables	EC tau-PET SUVR			ITC tau-PET SUVR			Meta-ROI tau-PET SUVR		
	Estimate	CI	*P-*value	Estimate	CI	*P-*value	Estimate	CI	*P-*value
*APOE* ɛ4 carriers									
FHS-CVD risk	0.01	−0.01 to 0.02	0.239	0.00	−0.01 to 0.01	0.793	0.00	−0.00 to 0.01	0.652
Sex (female)	−0.28	−0.81 to 0.25	0.294	−0.10	−0.42 to 0.23	0.541	−0.05	−0.32 to 0.22	0.714
Time lag	0.05	−0.03 to 0.13	0.200	−0.02	−0.06 to 0.03	0.490	−0.02	−0.06 to 0.02	0.402
Age	−0.00	−0.02 to 0.01	0.612	0.00	−0.00 to 0.01	0.845	0.00	−0.01 to 0.01	0.575
Aβ status (Aβ+)	0.19	−0.05 to 0.43	0.116	0.25	0.10–0.39	**0**.002***	0.21	0.08–0.33	**0**.001***
Education	0.02	−0.02 to 0.06	0.318	−0.01	−0.04 to 0.01	0.297	−0.01	−0.03 to 0.01	0.358
Sex (female × FHS-CVD risk	0.05	0.02–0.08	**0**.004***	0.02	0.00–0.04	**0**.049*	0.02	0.00–0.03	**0**.045*
*APOE* ɛ4 non-carriers									
FHS-CVD risk	0.00	−0.01 to 0.01	0.499	−0.00	−0.01 to 0.01	0.992	−0.00	−0.01 to 0.01	0.729
Sex (female)	0.18	−0.16 to 0.52	0.304	0.15	−0.10 to 0.39	0.242	0.07	−0.12 to 0.27	0.452
Time lag	0.00	−0.04 to 0.05	0.887	−0.01	−0.04 to 0.02	0.561	−0.01	−0.04 to 0.01	0.351
Age	0.01	−0.00 to 0.02	0.103	0.00	−0.01 to 0.01	0.821	0.00	−0.00 to 0.01	0.339
Aβ status (Aβ+)	0.08	−0.06 to 0.23	0.268	0.16	0.05–0.26	**0**.004***	0.14	0.05–0.22	**0**.002***
Education	0.00	−0.02 to 0.03	0.739	0.01	−0.01 to 0.03	0.170	0.01	−0.01 to 0.03	0.185
Sex (female) × FHS-CVD risk	0.00	−0.01 to 0.02	0.617	0.00	−0.01 to 0.01	0.998	0.00	−0.01 to 0.01	0.615

Results from linear regression analysis on *APOE* ɛ4 stratified groups revealed a significant interaction effect of sex and FHS-CVD risk on tau deposition in the EC, ITC and meta-ROI only among *APOE* ɛ4 carriers, after adjusting for the time between FHS-CVD risk assessment and tau-PET scan, age, Aβ status and education. Significant *P-*values are presented in bold, where *P < 0.05, **P < 0.01, ***P < 0.005, *P* < 0.001.Aβ = amyloid-β; CI = confidence interval; EC = entorhinal cortex; FHS-CVD risk = Framingham Heart Study cardiovascular disease risk; ITC = inferior temporal cortex; SUVR = standardized uptake value ratio.

**Table 7:** Main effect of cardiovascular disease risk on cortical tau deposition in female *APOE* ɛ4 carriers but not male carriers, after additionally adjusting for age, Aβ status and education

Predictor variables	EC tau-PET SUVR			ITC tau-PET SUVR			Meta-ROI tau-PET SUVR		
	Estimate	CI	*P-*value	Estimate	CI	*P-*value	Estimate	CI	*P-*value
Female *APOE* ɛ4 carriers									
FHS-CVD risk	0.06	0.03–0.09	**0**.002***	0.02	−0.00 to 0.04	0.052	0.02	0.00–0.03	**0**.028*
Time lag	0.03	−0.07 to 0.12	0.549	−0.03	−0.09 to 0.03	0.293	−0.03	−0.08 to 0.02	0.180
Age	−0.02	−0.06 to 0.02	0.279	−0.00	−0.03 to 0.02	0.811	−0.00	−0.02 to 0.02	0.734
Aβ status (Aβ+)	0.27	−0.10 to 0.63	0.138	0.33	0.11–0.56	**0**.005**	0.28	0.11–0.46	**0**.003***
Education	0.03	−0.04 to 0.11	0.364	0.01	−0.04 to 0.05	0.809	0.00	−0.04 to 0.04	0.952
Male *APOE* ɛ4 carriers									
FHS-CVD risk	0.00	−0.01 to 0.02	0.675	−0.00	−0.01 to 0.00	0.434	−0.00	−0.01 to 0.00	0.568
Time lag	0.11	−0.08 to 0.30	0.227	0.04	−0.06 to 0.15	0.409	0.04	−0.05 to 0.13	0.389
Age	−0.00	−0.03 to 0.03	0.964	0.00	−0.01 to 0.02	0.797	0.00	−0.01 to 0.02	0.625
Aβ status (Aβ+)	0.07	−0.30 to 0.43	0.703	0.10	−0.11 to 0.30	0.340	0.08	−0.10 to 0.26	0.357
Education	0.00	−0.06 to 0.06	0.893	−0.03	−0.06 to 0.00	0.067	−0.02	−0.05 to 0.01	0.143

Results from linear regression analysis on sex-stratified groups of *APOE* ɛ4 carriers revealed a significant main effect of FHS-CVD risk on tau deposition in the EC and meta-ROI only among female *APOE* ɛ4 carriers, after adjusting for the time between FHS-CVD risk assessment and tau-PET scan, age, Aβ status and education. Significant *P-*values are presented in bold, where *P < 0.05, **P < 0.01, ***P < 0.005, ****P < 0.001. Aβ = amyloid-β; CI = confidence interval; EC = entorhinal cortex; FHS-CVD risk = Framingham Heart Study cardiovascular disease risk; ITC = inferior temporal cortex; SUVR = standardized uptake value ratio.

## Discussion

In this study of CN older adults, we found that sex and *APOE* ɛ4 status modified the association between cardiovascular risk and tau deposition. After stratifying our sample by *APOE* ɛ4 status, we found that sex modified the association between cardiovascular risk and tau burden, among *APOE* ɛ4 carriers only. Finally, sex-stratified analyses showed that higher cardiovascular risk was associated with higher levels of EC, ITC and meta-ROI tau among female *APOE* ɛ4 carriers but not male carriers. Adjusting for additional covariates such as age, Aβ status and education attenuated interaction effects of sex, *APOE* ɛ4 and cardiovascular risk on tau deposition in the ITC and meta-ROI, but not the EC. The absence of a three-way interaction effect in the ITC and meta-ROI appears to be due to a more robust main effect of Aβ status on tau deposition in these regions among both *APOE* ɛ4 carriers and non-carriers. There was no such effect on tau deposition in the EC, which has been shown to be associated with *APOE* ɛ4, independently of Aβ burden.^49^ Furthermore, a recent study on CN older adults found that the interaction between higher cardiovascular risk and elevated Aβ burden was associated with higher levels of tau deposition in the ITC but not the EC.^50^ Despite the strong main effect of Aβ status on ITC and meta-ROI tau among *APOE* ɛ4 carriers and non-carriers alike, two-way interactions between female sex and cardiovascular risk on tau deposition were seen only among *APOE* ɛ4 carriers. Therefore, studies on larger samples should examine sex differences in the association between cardiovascular risk and tau deposition among groups stratified by both *APOE* ɛ4 and Aβ status.

Our findings are in line with other studies demonstrating stronger predictive relationships between vascular risk factors such as elevated plasma lipids and hypertension and incident Alzheimer’s disease in women compared with men,^1,51^ and are consistent with our prior study demonstrating that among community-dwelling older adults, higher FHS-CVD risk scores were associated with greater executive function and memory decline among women but not men, despite higher risk scores among men.^29^ Tau pathology is strongly correlated with clinical symptomatology,^32^ and recent evidence suggests that associations between cerebrovascular function and cognition are mediated by tau pathology.^12,13^ Therefore, the exacerbated effect of cardiovascular disease risk on cognition and clinical outcomes observed among women may be, at least partly, due to elevated tau deposition.

While our study was not designed to probe mechanisms of this effect, there are potential explanations, including the impact of hormonal changes in post-menopausal women: The abrupt decrease of oestrogen levels in post-menopausal women is associated with reduced glucose tolerance, as well as increased blood pressure, endothelial dysfunction and vascular inflammation.^52^ Oestrogen-binding on endothelial and vascular smooth muscle cells has been shown to prevent neointimal responses to acute vascular injury, resulting from atherosclerosis, hypertension and other vascular diseases.^53,54^ Therefore, the absence of the aforementioned vaso- and neuroprotective effects of oestrogen in post-menopausal women may contribute to a heightened inflammatory response to vascular risks, potentially leading to worse brain outcomes in women. Evidence of a more direct link between oestrogen and tau comes from animal studies showing that oestrogen depletion can lead to an increase in enzymatic activity (including protein kinase A and glycogen synthase kinase 3-β) involved in tau hyperphosphorylation.^55^

We found that sex differences in the association between cardiovascular risk and tau deposition were restricted to *APOE* ɛ4 carriers. This finding is consistent with a recent study showing that higher vascular risk and *APOE* ɛ4 interactively predicted memory decline in women but not men.^22^ Our results extend prior evidence of a stronger *APOE* ɛ4 effect on tau pathology in women,^24–27^ and introduce the role of cardiovascular disease risk as a potential factor contributing to this female *APOE* ɛ4-related susceptibility to tauopathy. Notably, *APOE* ɛ4 confers greater risk of developing MCI in women specifically between 55 and 70 years of age, compared with men.^56^ In this study, we found an effect of cardiovascular disease risk on tau deposition only among female *APOE* ɛ4 carriers, who were between 55 and 78 years of age. It is conceivable that the first two decades following menopause could be a critical time-window during which elevated cardiovascular vulnerability may predispose female *APOE* ɛ4 carriers to worse pathological and clinical outcomes.

Although the mechanisms of the interaction between *APOE* ɛ4 and vascular risk and its exacerbated impact on brain health among women remains unknown, the influence of sex hormones may be relevant. Oestrogen has been shown to inhibit microglial activation in response to acute inflammatory stimuli, thereby reducing the production of reactive oxygen species and proinflammatory molecules such as matrix metalloproteinase 9.^57^ However, the presence of an *APOE* ɛ4 allele inhibits the anti-inflammatory effects of oestrogen on microglial and peripheral microphage activation,^58^ suggesting that female *APOE* ɛ4 carriers are less able to benefit from the anti-inflammatory properties of circulating oestrogen, compared with non-carriers. Conversely, testosterone, which has similar anti-inflammatory properties, has also been shown to interact with *APOE* ɛ4 to impact cognitive function and Alzheimer’s disease risk.^59–61^ Animal studies showed that *APOE* ɛ4 can reduce androgen receptor levels in the brain, suggesting that *APOE* ɛ4 carriers may be particularly vulnerable to the effects of lower testosterone.^61^ Sundermann *et al.*^62^ found that the association between lower testosterone and higher levels of cerebrospinal fluid phosphorylated tau were strongest among female *APOE* ɛ4 carriers. Consequently, the heightened vascular inflammation seen in post-menopausal women along with the heightened cerebrovascular impact of *APOE* ɛ4 in the absence of the neuroprotective effects of sex hormones may interact to exacerbate the effects of cardiovascular risk factors on Alzheimer’s disease tauopathy in female *APOE* ɛ4 carriers. Future experimental research is needed to probe this possible explanation for our findings.

### Limitations

The comparatively higher mortality rates at younger ages due to cardiovascular disease in men may have resulted in a survival bias in our study. This survival bias along with women’s resilience against cardiovascular disease up until mid-life and heightened vascular vulnerability after menopause may lead to greater vascular and Alzheimer’s disease comorbidity among women. Given that participants with a Hachinski ischaemic score >4 are excluded from the ADNI study, our sample was composed of participants in relatively good cardiovascular health. Larger studies in samples that are more representative of cardiovascular health in the aging population are needed to investigate potential sex-disparities further. ADNI also does not provide details on women’s health such as age at menopause and exposure to female-specific cardiovascular risk factors such as pre-eclampsia, which could be critical to the interpretation of our findings. Furthermore, since our sample was predominantly composed of non-Hispanic white men and women, our results may not generalize to other racial and ethnic groups. Studies have shown that risk factors and cardiovascular disease manifestations can differ significantly between racial groups,^63^ highlighting the importance of studying racially and ethnically diverse samples. Another limitation of our study was its small sample size, which may have particularly affected the performance of tau deposition models in male and female *APOE* ɛ4 carriers (*n* = 23 and *n* = 24, respectively). Nevertheless, given the similar numbers of *APOE* ɛ4 carriers among men and women in the study, it is probably unlikely that the effect seen in women was driven by greater statistical power. Finally, the cross-sectional nature of the study along with the time lag between cardiovascular risk and tau-PET assessments limits our ability to draw conclusions about the temporality of associations between cardiovascular disease and tau deposition. Therefore, future longitudinal studies are needed to examine the extent to which sex and *APOE* ɛ4 impact the association of cardiovascular risk and accumulation of tau over the course of Alzheimer’s disease.

## Conclusion

Our results suggest that female *APOE* ɛ4 carriers may be particularly vulnerable to the impact of cardiovascular risk on cortical tau deposition, despite having lower cardiovascular risk scores than men. Our findings are clinically relevant, given the modifiable nature of many cardiovascular risk factors such as smoking and high blood pressure, and may contribute to our understanding of the observed heightened susceptibility to tauopathy among female *APOE* ɛ4 carriers. If replicated in larger, population-based samples, these findings could have important implications for the treatment of even low levels of vascular risk in seemingly healthy CN older women who have a genetic risk for Alzheimer’s disease. Future studies exploring associations between cardiovascular risk and Alzheimer’s disease -related outcomes should consider stratifying by sex and *APOE* ɛ4.

## Abbreviations

ADNI =: Alzheimer’s Disease Neuroimaging Initiative
Aβ =: amyloid-β
CDR =: Clinical Dementia Rating
CDR-GS =: Clinical Dementia Rating global score
CN =: cognitively normal
EC =: entorhinal cortex
FHS-CVD risk =: Framingham Heart Study cardiovascular disease risk
FTP =: Flortaucipir
ITC =: inferior temporal cortex
ROI =: region of interest
SUVR =: standardized uptake value ratio
